# Phase-contrast neutron imaging compared with wave propagation and *McStas* simulations

**DOI:** 10.1107/S1600576724003030

**Published:** 2024-05-10

**Authors:** Estrid Buhl Naver, Mads Bertelsen, Maja Østergaard, Domenico Battaglia, Peter Willendrup, Pavel Trtik, Søren Schmidt, Henrik Birkedal, LuiseTheil Kuhn

**Affiliations:** a Department of Energy Conversion and Storage, Technical University of Denmark, Fysikvej 310, Kongens Lyngby, Denmark; b European Spallation Source ERIC, PO Box 176, Lund, Sweden; c Department of Chemistry and iNANO, Aarhus University, Gustav Wieds Vej 14, Aarhus, Denmark; d Department of Physics, Technical University of Denmark, Fysikvej 307, Kongens Lyngby, Denmark; e Laboratory for Neutron Scattering and Imaging, Paul Scherrer Institut, Villigen, Switzerland; Australian Centre for Neutron Scattering, ANSTO, Australia

**Keywords:** phase-contrast imaging, neutron imaging, simulations, refraction, *McStas*

## Abstract

Propagation-based phase contrast, for example in the form of edge enhancement contrast, is well established within X-ray imaging but is not widely used in neutron imaging. This technique can help increase the contrast of low-attenuation samples but may confuse quantitative absorption measurements. Therefore, it is important to understand the experimental parameters that cause and amplify or dampen this effect in order to optimize future experiments properly. Two simulation approaches have been investigated, a wave-based simulation and a particle-based simulation conducted in *McStas* [Willendrup & Lefmann (2020). *J. Neutron Res.* **22**, 1–16], and they are compared with experimental data. The experiment was done on a sample of metal foils with weakly and strongly neutron absorbing layers, which were measured while varying the rotation angle and propagation distance from the sample. The experimental data show multiple signals: attenuation, phase contrast and reflection. The wave model reproduces the sample attenuation and the phase peaks but it does not reproduce the behavior of these peaks as a function of rotation angle. The *McStas* simulation agrees better with the experimental data, as it reproduces attenuation, phase peaks and reflection, as well as the change in these signals as a function of rotation angle and distance. This suggests that the *McStas* simulation approach, where the particle description of the neutron facilitates the incorporation of multiple effects, is the most convenient way of modeling edge enhancement in neutron imaging.

## Abstract 2

This article discusses the phase-contrast effect and compares two simulation methods based on different theoretical frameworks. It demonstrates the ability to reproduce the phase effect in experimental data, using the well known simulation framework *McStas* [Willendrup & Lefmann (2020). *J. Neutron Res.* **22**, 1–16].

## 1. Introduction

Neutron imaging is a useful tool for investigating the multi-scale structure of different types of materials for both biological and technical applications. This is due to the good penetration capabilities of neutrons and the non-continuous dependence of the interaction cross section with atomic number (Kardjilov *et al.*, 2018 ▸; Strobl *et al.*, 2009*b* ▸). The primary contrast method for neutron imaging is by attenuation contrast, but this has limitations when investigating materials with low or very similar neutron attenuation. One way to solve this challenge is by measuring the phase difference introduced by the variation in refractive index of the sample.

There are multiple ways of performing phase-contrast imaging, either by interferometry (Kim *et al.*, 2019 ▸; Pushin *et al.*, 2017 ▸) or by propagation-based phase contrast (Allman *et al.*, 2000 ▸; Paganin *et al.*, 2023 ▸). The benefit of the latter is that no gratings are necessary to measure the phase shift. For this reason, propagation-based imaging is very powerful and widely used in X-ray imaging (Snigirev *et al.*, 1995 ▸; Alloo *et al.*, 2022 ▸; Bidola *et al.*, 2017 ▸). It has also been exploited in neutron imaging, where it has been used to detect cracks in aluminium (Fiori *et al.*, 2006 ▸) and investigate pores in bone (Østergaard *et al.*, 2023*a* ▸,*b* ▸).

Propagation-based phase contrast relies on differences in the refractive index resulting in changes in the wavefront upon passing through the sample, which leads to interference and a phase-contrast signal appearing when increasing the propagation distance of the neutrons between the sample and the detector. This requires a partially coherent beam (Lehmann *et al.*, 2017 ▸).

When paired with phase retrieval, noise can be decreased and contrast enhanced (Paganin *et al.*, 2023 ▸), and similarly for white-beam measurements (Østergaard *et al.*, 2023*a* ▸). However, if quantification of attenuation is important, phase contrast can be a hindrance, especially at the edges of the sample.

For optimal experimental design, it is important to take phase-contrast effects into account, either enhancing or suppressing them depending on the needs of the experiment. Studies have investigated how different experimental parameters influence the phase-contrast effect (Strobl *et al.*, 2009*a* ▸; Lehmann *et al.*, 2017 ▸) on simple metal samples. However, for more complicated samples, it would be beneficial to compare with simulations. The resulting question that arises is how best to simulate this effect. In X-ray imaging the wave model of propagation is widely used to describe phase contrast (Paganin, 2006 ▸) but for neutron imaging only ray-tracing has been performed so far. Butler & Lehmann (2013 ▸) and Lehmann *et al.* (2017 ▸) both implemented ray-tracing models based on Snell’s law and simulated single-material samples. They obtained good qualitative agreement between the models and the experimental data. The samples of Lehmann *et al.* (2017 ▸) are simple single-material samples simulated in a vacuum. In the present paper a multi-material sample was designed, consisting of metal foil layers of various widths, and experiments were performed on the imaging beamline BOA at the Paul Scherrer Institute. This experiment was simulated using both a particle and a wave description of the neutron in order to compare which approach is most convenient to describe such experiments.

## 2. Methods

### 2.1. Neutron imaging of metal foil sample

**Figure 1:**
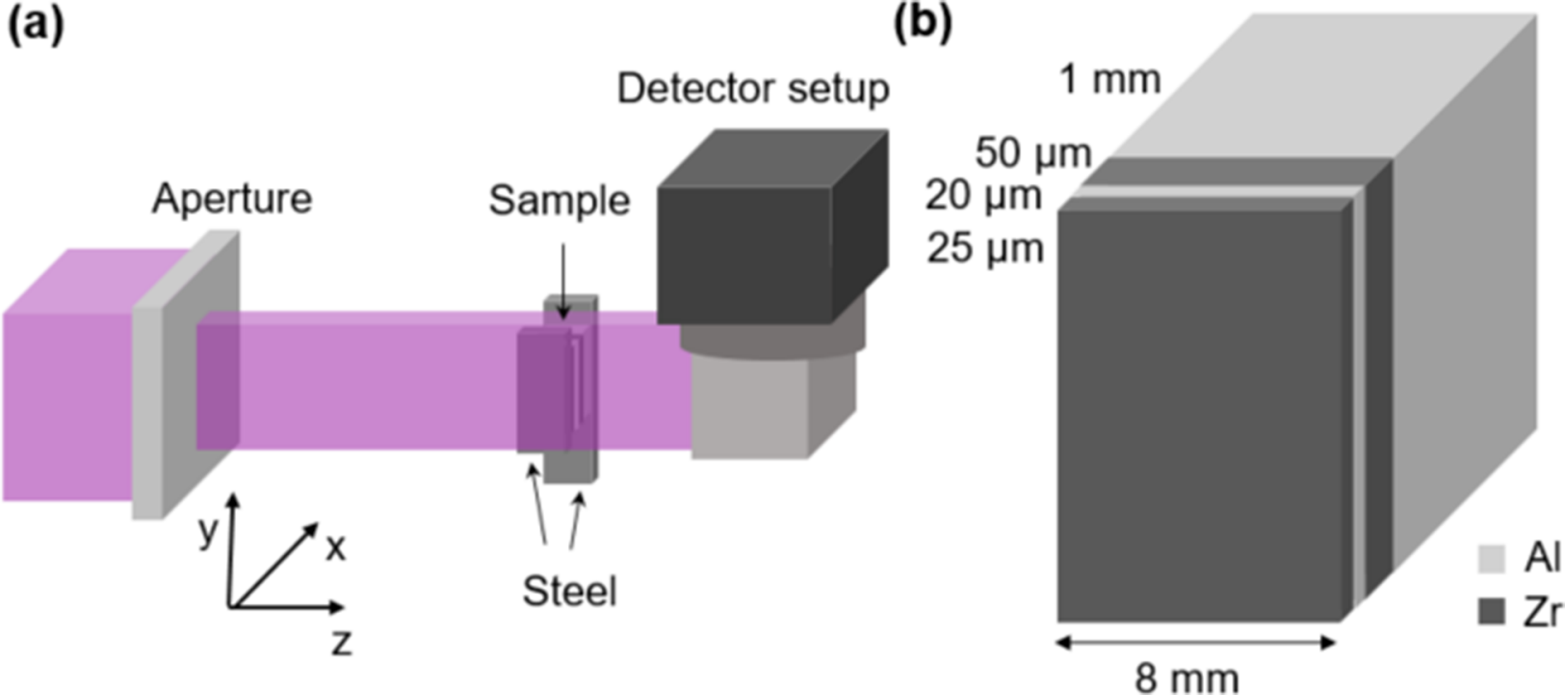
(*a*) A sketch of the experimental setup for phase-contrast neutron imaging, showing the metal foil sample sandwiched between two steel layers. (*b*) A sketch of the metal foil sample without the steel layers. Note that the sample is not to scale.

A metal foil sample was designed for a high-phase-contrast signal and a simple geometry. This consisted of Al and Zr sheets (coherent scattering cross section and thermal neutron absorption cross section of 6.44 and 0.185 barn, respectively, for Zr and 1.495 and 0.231 barn, respectively, for Al) with thicknesses of 1 mm, 50 µm, 20 µm and 25 µm, as seen in Fig. 1 ▸(*b*). These were mounted between two layers of 316 stainless steel of thickness 1.5 mm. Al and Zr were chosen for their low attenuation and high coherent scattering, in order to ensure that the contrast between the Al and Zr foils would be due to the phase and not to attenuation differences. The steel was added because of its high attenuation which helped align the sample with the beam. The layer thicknesses were chosen to test the resolution of the measurements, with the expectation that the 1 mm and 50 µm layers would be easily resolved and resolving the 20 µm and 25 µm layers would be more challenging. This enabled us to investigate whether phase contrast could be used to push the limit of the neutron imaging resolution.

Neutron imaging experiments on the metal foil sample were performed on the BOA beamline at the Paul Scherrer Institute (Morgano *et al.*, 2014 ▸). The beam was modified with a rectangular aperture of 10 × 120 mm^2^ to increase coherence in the horizontal direction. The wavelength range of the beam was 0.8–10 Å, with a weighted mean wavelength of 3.8 Å. A high-resolution neutron imaging detector (Trtik & Lehmann, 2016 ▸), equipped with an isotopically enriched 157-gadolinium oxysulfide scintillator (Crha *et al.*, 2019 ▸) and CCD camera (iKon-L, Andor), was utilized for the experiment, yielding images of 2.7 µm pixel size. The distance between the aperture and the detector was 5.895 m, resulting in *L*/*D* = 589.5 in the horizontal direction. The measurements were taken at four sample-to-detector distances and at different rotation angles to find the position at which the beam was closest to parallel with the foil layers. The sample was measured at distances of *l* = 5, 25, 35 and 40 mm. A sketch of the setup is shown in Fig. 1 ▸. For each distance the rotation was varied in steps of 0.02° over a range of 1°. The specific angle range where the sample was closest to parallel with the beam is shown in Table 1 ▸. For each distance and angle, five radiographs were acquired with exposure times of 120 s each.

**Figure 2:**
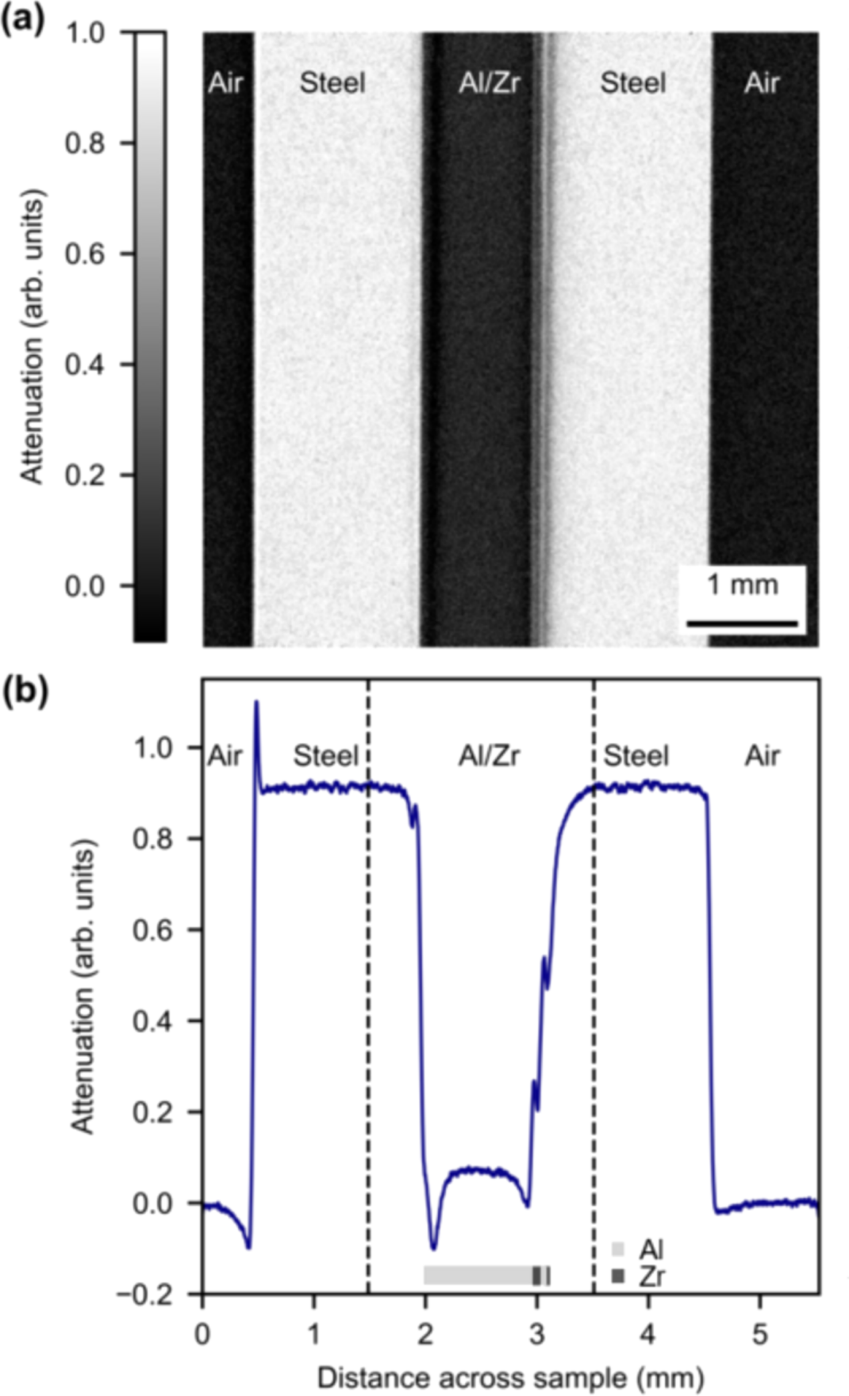
(*a*) The radiograph and (*b*) the average attenuation of the radiograph of a metal foil sample sandwiched between plates of steel at *l* = 25 mm, after flat- and dark-field correction. The vertical dashed lines in panel (*b*) show the region of interest (ROI) in the data. The inset in (*b*) shows the placement of the Al and Zr layers.

The collected data were flat- and dark-field corrected with software by Kaestner (2017 ▸) and spots from γ-radiation were removed. Afterwards, the data were summed in the vertical direction to increase statistics and enhance the signal. An example radiograph of the sample is shown in Fig. 2 ▸(*a*).

### 2.2. Simulation methods

#### 2.2.1. Wave simulation

We have developed a wave-propagation simulator based on the Fresnel diffraction integral (Als-Nielsen & McMorrow, 2011 ▸). This was implemented in Python in two parts: (i) the neutron–sample interaction and (ii) the wavefront propagation after the sample.

The sample is defined as an array of pixels which are filled with a medium with refractive index (Treimer *et al.*, 2005 ▸) 

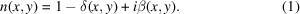

Here δ = *b*_coh_ρλ^2^/(2π) and β = σ_att_ρλ/(4π), where *b*_coh_ is the coherent neutron scattering length, σ_att_ = σ_abs_ + σ_incoh_ is the sum of the absorption and incoherent cross sections, ρ is the atomic density of the material, and λ is the neutron wavelength. The coordinates (*x*, *y*) define the position in the plane perpendicular to the beam axis *z* as defined in Fig. 1 ▸(*a*).

We assume a projection approximation (Morgan *et al.*, 2010 ▸), which means that the neutron beam is assumed to have no propagation inside the object, *i.e.* the sample is considered to be very thin. This can be described as the sample adding a constant phase shift as described by equation (2),

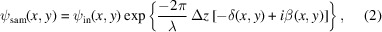

where Δ*z* is the thickness of the sample along the beam direction and ψ_in_ is a wavefunction describing the incoming wave.

After exiting the sample, the wave is propagated a distance *l* along the *z* axis using the Fresnel propagator in Fourier space, 

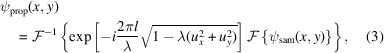

where 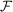 and 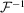 are the Fourier and inverse Fourier transforms, respectively, and *u*_*x*_, *u*_*y*_ are the spatial frequencies.

As a starting point, this simulation assumes a perfectly monochromatic and coherent beam. To simulate a white beam, multiple waves were generated, propagated and added together in a weighted sum. The neutron refractive index at different wavelengths was calculated with the use of wavelength-dependent neutron cross sections from Los Alamos T-2 Nuclear Information Service (Chadwick *et al.*, 2011 ▸; https://t2.lanl.gov/nis/data/endf/endfvii.1-n.html). To model the effect of beam divergence, the resulting image was convoluted with a Gaussian function with a standard deviation σ based on how many pixels were spanned by the geometric blur *d*, 

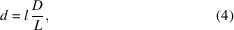

where *l* is the sample-to-detector distance, *D* is the width of the aperture and *L* is the aperture-to-sample distance. The geometric blur was assumed to span three standard deviations. This leads to the standard deviation of the Gaussian blur being defined as σ = *d*/6.

When simulating a polychromatic beam using 20 wavelengths from the BOA spectrum and applying the blur from the beam divergence, the simulated phase signal disappeared completely. This suggests that the assumption behind this implementation of the polychromatic beam is flawed, namely the assumption that the white beam behaves like a series of discrete monochromatic beams. In an experiment the neutrons of different wavelengths can interfere with each other, and this is not replicated when propagating the individual wavelengths separately. For this reason we chose to simulate a simpler model, which is a divergent monochromatic beam with a wavelength matching the mean wavelength of the BOA spectrum, 3.8 Å. The wave simulation results presented here are all simulated with this monochromatic beam.

The simulated parameters used for the experiment and the material constants used for the sample are seen in Tables 1 ▸ and 2 ▸, respectively.

#### 2.2.2. *McStas* simulation

*McStas* (Willendrup & Lefmann, 2020 ▸) is a simulation program that can be applied to Monte Carlo ray tracing of neutron experiments. The neutrons are represented semi-classically by simultaneously well defined position, velocity, time and spin vectors. In order to simulate realistic values for neutron intensities, the simulated neutron rays represent multiple neutrons using a weight factor. The intensity of the individual ray is then updated as it interacts with the various parts of the instrument. The simulations are modular, consisting of components that correspond to parts of the instrument like beam guides, slits, samples and detectors.

The sample was implemented using the *McStas Union* framework (Bertelsen, 2017 ▸) as a collection of metal foils, as shown in Fig. 1 ▸(*b*). The *Union* geometry was modified to include refraction and reflection at material surfaces. Both effects are modeled as changes in direction of individual neutron rays, with the refraction angle θ_2_ defined by Snell’s law: 

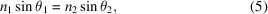

where *n*_1_ and *n*_2_ are the refractive indices of two neighboring materials, and θ_1_ is the incidence angle of the beam.

The refractive index of the neutron is calculated as (Sears, 1982 ▸) 

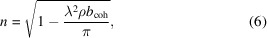

where λ is the wavelength of the incoming neutron, ρ is the atomic density of the sample and *b*_coh_ is the coherent neutron scattering length as calculated from the coherent scattering cross section.

The benefit of defining the sample in *Union* is that the neutron can interact with the different parts of the sample in any order, instead of in the order in which they are defined, and we can model air around the sample instead of vacuum.

In addition to refraction and reflection, the sample can also absorb and scatter neutrons. This is handled as a separate probability, based on the neutron absorption and scattering cross sections of the material. For the scattering mechanism, both incoherent and coherent scattering are included. The transmission probability of the material over a path length *r* is (Bertelsen, 2017 ▸) 

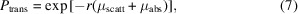

where μ_scatt_ and μ_abs_ are the inverse penetration depths for scattering and absorption, respectively. These inverse penetration depths are calculated from the cross section multiplied by the number of atoms per unit volume.

The simulation consisted of the BOA instrument, developed and benchmarked during the SINE 2020 project (https://github.com/matteobsu/AMG-Beamlines-McStas), and the sample made using *McStas* *Union* components. The BOA instrument was set to have the same slit configuration and detector size as the experiment. The parameters used to model the experiment and sample are shown in Tables 1 ▸ and 2 ▸, respectively. Using the BOA instrument model results in a precise model of the beam divergence and wavelength distribution. However, the resolution of the simulated data is better than that of the experimental data. The lower experimental resolution could be due to sample or detector vibrations, or blur caused by the scintillator thickness, which are not included in the *McStas* model. To model these effects and match the resolution of the data, the data were blurred using a Gaussian function with a standard deviation of five pixels.

## 3. Results and discussion

### 3.1. Experimental data

Fig. 2 ▸(*a*) shows a radiograph of the metal foil sample sandwiched between two steel layers. It is clearly seen that the steel absorbs significantly more than the metal foil sample, and it is possible to see lines from the thin foil layers at the left edge of the right-hand steel layer. To obtain the sample profile, an average is performed in the *y* direction and the result is shown in Fig. 2 ▸(*b*). The peaks at the interfaces with the left-hand piece of steel and between the Al/Zr layers are clearly seen. There are no peaks visible at the right steel/air interface on this figure. This is due to the misalignment between the right-hand steel surface and the beam at the plotted angle, which shows that the material surfaces of the sample are not completely parallel. The data show that the different materials give rise to different peak heights due to the relative differences in coherent scattering. The interfaces with the largest phase peaks are steel/air and steel/Al. In contrast, the Al/Zr interfaces give rise to smaller peaks. The region of interest (ROI) is outlined by the two dashed lines.

**Figure 3:**
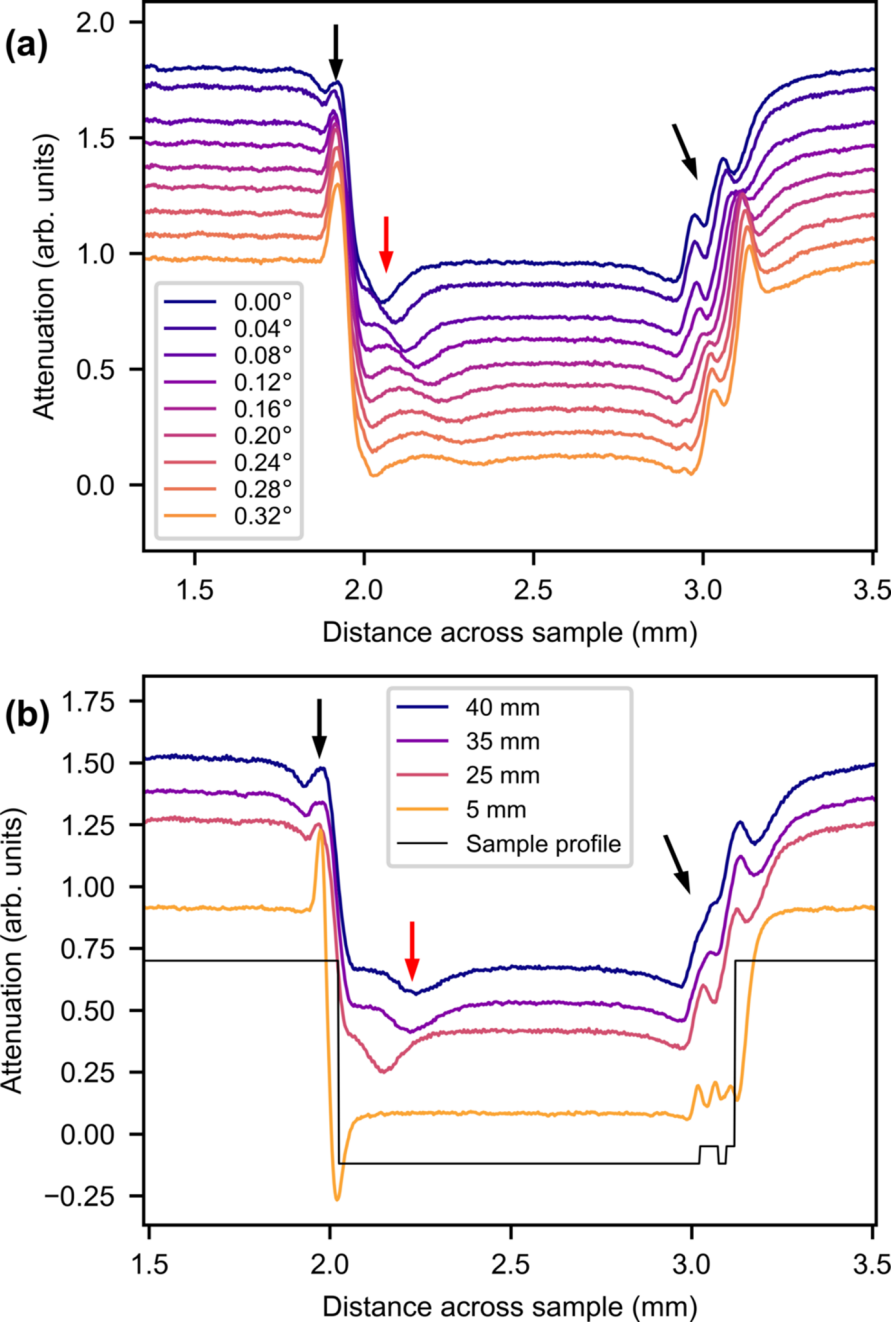
Plotted ROIs of average attenuation for (*a*) nine different angles at *l* = 25 mm and (*b*) four different sample-to-detector distances at an angle of 0.02°. The sample profile is plotted in (*b*). The graphs are offset vertically. Black arrows indicate phase-contrast peaks and red arrows indicate reflection peaks.

To investigate the behavior of the signal transmitted through the metal foils, the ROI of the average attenuation was plotted as a function of rotation angle in Fig. 3 ▸(*a*) and of sample-to-detector distance in Fig. 3 ▸(*b*). In both figures the graphs are cropped to show only the metal foil sample and part of the steel layers.

There are multiple features in the data. It is again seen that the steel layers to the sides have a higher absorption than the Al and Zr foils in the middle. There is a large peak to the left from the left-hand steel layer, marked by a black arrow, with a smaller neighboring peak marked by a red arrow. The peak marked by the red arrow moves and flattens out as the rotation angle increases and the distance increases. There are two smaller peaks to the right of the sample from the thin Zr and Al layers. The heights of the peaks increase significantly between 5 and 25 mm, suggesting that the peaks stem from phase contrast. This is consistent with the expectation of phase contrast being necessary to distinguish Al and Zr due to their low attenuation. The change in peak position as a function of angle is consistent with the assumption of phase contrast.

The peak marked by the red arrow both moves and flattens more than the peaks with black arrows. This could be because this is a reflection off the steel layer, whereas the black peaks are phase-contrast peaks that are less sensitive to the exact rotation of the sample.

All of the peaks broaden as the distance increases due to the divergence of the beam.

There are peaks missing from the Al/Zr interfaces and Zr/steel interface to the right. Four peaks are expected to the right, corresponding to the four interfaces. Of these, only three are seen at 5 mm and two at 25 mm and higher. The missing peak at 5 mm is probably due to misalignment of the foil layers; if they are not completely parallel the phase peaks will show up at different rotation angles. This is seen for the right-hand steel/air interface, which is at a different rotation angle from the left-hand steel/air peak. The decrease in the number of peaks between 5 and 25 mm is a result of blur from the divergence of the beam washing out the third peak.

### 3.2. Wave simulation data

**Figure 4:**
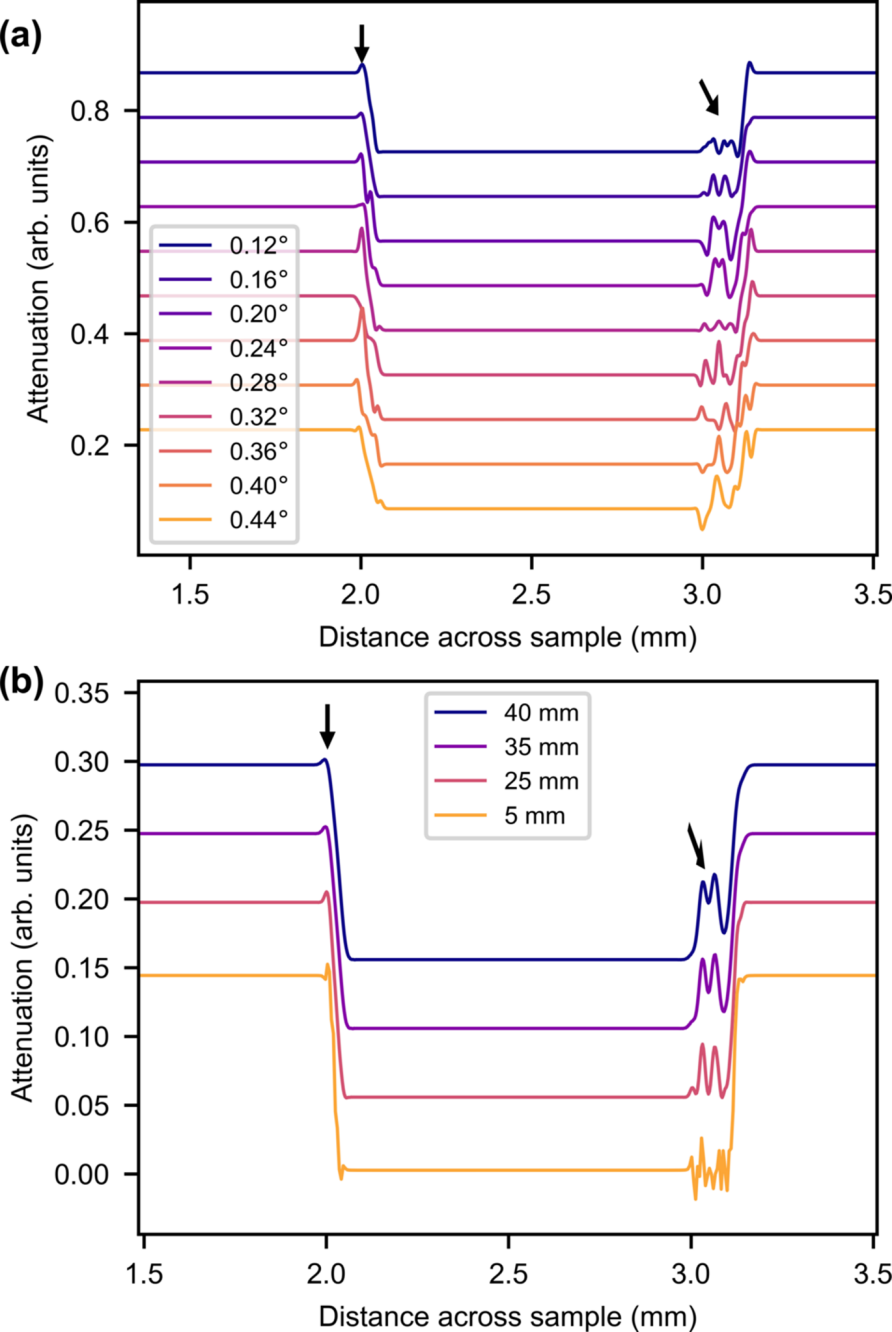
ROIs of average attenuation of wave-simulated radiographs for (*a*) different rotation angles at *l* = 25 mm and (*b*) different sample-to-detector distances at an angle of 0.16°. The graphs are offset vertically. Black arrows indicate phase-contrast peaks.

The simulation results are shown in Fig. 4 ▸. The steel is shown to absorb more than the Al and Zr foils, as in the experimental data, but the phase-contrast peaks are relatively smaller.

As in the experimental data, there is a peak from the steel layer and peaks from the thin Zr and Al layers, marked by arrows, but the behavior of the peaks is different from that of the experimental data. Fig. 4 ▸(*a*) shows that the left-hand peak from the steel changes size and becomes periodically smaller and larger with increasing rotation angle. This periodic behavior is not consistent with the experimental data and is probably due to phase wrapping, as the rotation angle changes the amount of material the beam travels through. The right-hand peaks change size according to the angle of rotation, in a seemingly random fashion. This could be due to phase wrapping and could also be influenced by attenuation changes.

The peak marked with a red arrow in Fig. 3 ▸ is entirely missing in this simulation. This supports the theory of this being a reflection peak, as reflection effects are not included in the wave simulation.

The peak behavior as a function of propagation distance in Fig. 4 ▸(*b*) shows that the steel peak increases in height with increasing distance. This is expected for a phase-contrast peak. The Zr/Al peaks also increase in height and become more blurry with distance. This is consistent with the experimental data.

### 3.3. *McStas* simulation data

**Figure 5:**
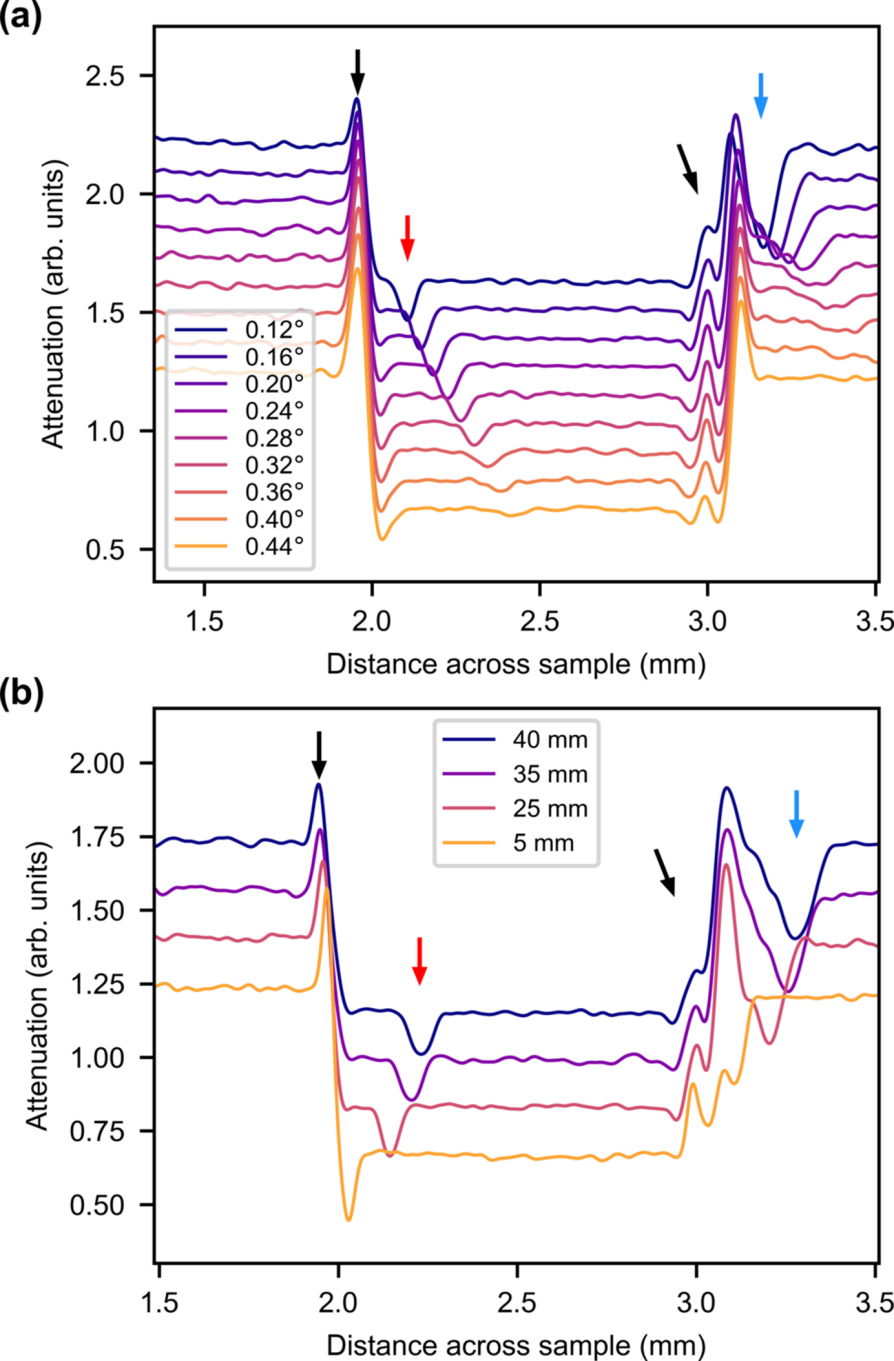
ROIs of average attenuation of *McStas*-simulated radiographs for (*a*) different rotation angles at *l* = 25 mm and (*b*) different sample-to-detector distances at an angle of 0.16°. The graphs are offset vertically. Black arrows indicate phase-contrast peaks, red arrows indicate reflection peaks from steel and blue arrows indicate reflection peaks from Al.

The *McStas* results are shown in Fig. 5 ▸ and they correspond fairly well to the experimental data. Both the steel reflection (red arrow) and phase-contrast (black arrow) peaks of the steel layer to the left are reproduced.

The angular and distance dependences of the simulated reflection resemble the experimental data. In the *McStas* simulation there is an additional reflection, marked with the blue arrow, which is assumed to be from one of the Zr or Al foil layers. This behaves similarly to the steel reflection, though it seems to vanish faster as a function of rotation angle. This reflection is probably absorbed by the right-hand steel layer in the experimental data.

The locations and behavior of the phase-contrast peaks resemble the experimental data with one exception: two peaks (at 3 mm across the sample) change in height and width with rotation angle and in width with the propagation distance. Three peaks in the experimental data (at *l* = 5 mm propagation distance) exhibit this tendency. A convolution of the phase peak and the Al reflection peak at *l* = 5 mm is observed. As the distance increases, the Al reflection moves right and the phase peak regains its height. This shows that the phase peaks do not change height as a function of distance in this simulation as they do in the experimental data.

## 4. Conclusion

In neutron imaging, there are several characteristics that affect contrast formation, the most common of which is attenuation. The phase changes introduced by the sample, by scattering and in some cases by reflections from sample edges can also show on the measured image.

Previously the phase change and reflection as a function of various parameters have been investigated (Strobl *et al.*, 2009*a* ▸; Lehmann *et al.*, 2017 ▸), and the ray-tracing description of refraction and reflection was found to be a good fit for the data. Phase changes have also been simulated using ray-tracing models on single-material samples (Butler & Lehmann, 2013 ▸; Lehmann *et al.*, 2017 ▸).

We aimed to test whether this was also the case for a more complex sample consisting of multiple layers with different attenuation and scattering cross sections. We have shown that a ray-tracing model in *McStas*, with the sample implemented in *Union*, replicates the data quite well, including attenuation, phase contrast and reflection. This confirms the conclusion of a ray-tracing model using Snell’s law as a good way of modeling neutron phase-contrast data.

The wave simulation showed the presence of phase peaks at the interfaces but did not reproduce the behavior of these peaks as a function of rotation angle. The discrepancy in rotation behavior for the peaks could be caused by discrepancies between the simulated beam and the experimentally measured beam and the assumption that the sample is very thin.

In the experimental data, the peaks do increase in height between *l* = 5 mm and *l* = 25 mm, but this is not replicated by the *McStas* simulations. This indicates that the ray-tracing description does not explain all effects in the experimental data.

In the future, if experiments are made with high neutron coherence, then the wave simulation might be more relevant for describing the experimental data.

## Figures and Tables

**Table 1:** Table of simulation and experimental parameters

	Experiment	Wave simulation	*McStas* simulation
Angle (°)	−1.42 to −1.10	0.12–0.44	0.12–0.44
*l* (mm)	5, 25, 35, 40	5, 25, 35, 40	5, 25, 35, 40
Aperture (mm)	120 × 10	–	120 × 10
Pixel (µm)	2.7	2.7	2.7
Spectrum (Å)	0.8–10	3.8	0.8–10

**Table 2:** Table of material parameters used for absorption, incoherent scattering and phase contrast in the wave and *McStas* simulations Neutron cross sections and scattering length values for individual elements are taken from NIST tables (Munter, 1992 ▸). The values for steel have been calculated as an average of the values for Fe, Cr and Ni, weighted with their concentrations in the alloy.

Variable	Al	Zr	Steel
Wave simulation			
σ_abs_ (barn)	0.231	0.185	2.87
σ_incoh_ (barn)	0.0082	0.02	1.22
*b*_coh_ (10^−15^ m)	3.45	6.44	8.56
ρ (10^28^ m^−1^)	6.0	4.3	9.0
			
*McStas* simulation			
μ_abs_ (m^−1^)	1.39	0.796	25.8
σ_incoh_ (barn)	0.0082	0.02	1.22
σ_coh_ (barn)	1.495	6.44	9.84
ρ_macro_ (g cm^−3^)	2.7	6.5	8.3
